# Dr. Fowler Bradnack

**Published:** 1891-06

**Authors:** 


Dr. Fowler Bradnack, who died in this city on Easter Sunday, March 29, 1891, was born at Great Yarmouth, Norfolk, England. He came to Buffalo when quite a young man, and by his winning manners, fine mind, and brilliant talents, won many friends.

Many will remember his humorous poems, published in several papers and magazines, and especially the one that gained for him the prize of life-membership of the Buffalo Library. He studied medicine, and graduated from the Buffalo Medical College in 1871 with distinguished honors.

He was married, in 1873, to Nettie L. Hickox, of Winfield, Herkimer county, N. Y., and removed to New York, where he practised his profession. He won the hearts of his patients by his kindness and sympathy, as well as by his thorough knowledge and skill.

The death of his wife, which occurred in 1880, was a severe blow to the doctor. Being a man of literary taste, he continued to write both prose and verse, but only occasionally publishing his writings. He married again in 1887, a Buffalo lady (who survives him), and returned to Buffalo.

Dr. Bradnack was a close student. Literature and music were his delight. Those who heard him play on the piano the classical music he so much loved, were charmed by his wonderful and delicate touch. Many a time had he turned to find his audience weeping. He was a frequent visitor at the Library, and, about a year ago, he delivered An Essay on Snakes, before the Buffalo Society of Natural Sciences. His unpublished manuscripts show years of thoughtful toil. His last published article was in the June and July number of the JOURNAL, the subject being Death, Its Modes, Signs and Premonitions. He made the remark, while writing it, "This is the last article I shall ever write!" feeling, no doubt, symptoms of the malady that sent him to an early grave.

He had a long and wasting illness, but his patience, and even cheerfulness, was very remarkable. He often spoke affectionately of his many friends, who were so untiring in their attentions. He leaves a father, Rev. Isaac Bradnack, of Panama, Chautauqua county, N. Y., and two sisters, Mrs. Elizabeth Wilkinson, of Panama, and Mrs. Dr. W. C. Earl, of this city.

