# Correction

**Published:** 1891-09

**Authors:** 


Correction.—In the obituary of the late Dr. Fowler Bradnack, published in the June number of the Journal, there is inaccuracy which we are pleased to correct. Dr. Bradnack was twice married; the second time to Mrs. Susie Bulson, of Buffalo, N. Y., this marriage having taken place in New York City, June 21, 1885.

